# Genomic Crosstalk Between Nuclear Receptors in Hormone-dependent Cancers

**DOI:** 10.1210/endocr/bqaf149

**Published:** 2025-10-28

**Authors:** Moray J Campbell

**Affiliations:** Board of Governors Innovation Center, Cedars-Sinai Medical Center, Los Angeles, CA 90048, USA; Cedars-Sinai Cancer, Cedars-Sinai Medical Center, Los Angeles, CA 90048, USA; Biomedical Sciences, Cedars-Sinai Medical Center, Los Angeles, CA 90048, USA

**Keywords:** nuclear receptors, cistrome, transcriptome, pioneer factors, SWI/SNF (BAF) coregulator complexes

## Abstract

Nuclear receptors (NRs) orchestrate transcriptional programs that regulate cell fate decisions, and when these processes are disrupted, they can drive hormone-dependent cancers. This review summarizes mechanisms by which NRs function collectively, or crosstalk, to bring about the complex transcriptional control of cell fate decisions and indicate where these processes can act as cancer drivers. These crosstalk mechanisms include the exchange of coregulators between NRs and as well as genomic convergence of NRs. Evidence is also discussed for how NRs potentially pass through a continuum of interactions as part of a biological ratchet mechanism to regulate gene transcription. In this continuum, pioneer factors drive chromatin competence for NRs and, along with mammalian SWI/SNF complexes, facilitate transient assisted loading between NRs, as well as more stable crosstalk in the form of mitotic bookmarking, which allows inheritance of transcriptional control. NR crosstalk is also sustained through the function of larger and perhaps more stable interactions, such as through the megatrans complex. Also considered to explain NR crosstalk is the established and emerging understanding of the grammar of motif selection, and this is placed in the context of NR network approaches, for example in breast cancer. Finally, a systems-level framework, called NuRome, is discussed that combines high-dimensional data at the cistrome, transcriptome, and proteome levels to provide a predictive understanding of NR crosstalk and transcription in cancer.

## The Nuclear Receptor Superfamily Exerts Widespread Control Over Cell Fate in Hormone-responsive Cancers

Sir George Beatson connected elevated hormone levels to breast cancer (BrCa) risk in the 19th century (1), and in the 1940s Charles Huggins and colleagues expanded the concept to prostate cancer (PCa) risk (2). These foundational studies catalyzed the field of how nuclear receptor (NR) signaling impacts hormone-dependent cancers.

Unbeknownst to Beatson and Huggins, their pioneering studies centered on steroid hormone type I NRs (Table 1), which in turn profoundly shaped the view of NR functions by the research community. In this view, a given NR is inactive in the cytoplasm and is activated by binding high-affinity ligand that in turn triggers receptor homodimerization and transport to the nucleus. Ligand binding also induces NR conformational changes that allow coactivator binding that catalyze enzymatic regulation of chromatin accessibility and gene expression. Whilst these details are largely true for type I NRs, they are neither shared by all NRs nor do they encapsulate a complete functional view that explains crosstalk within the NR superfamily.

**Table 1.** Summary of the nuclear receptor superfamily indicating the receptor gene name, the type, the recommended protein name, and canonical ligands, if known

Gene name	Subfamily	Recommended protein name	Canonical ligand
NR3A1/ESR1	Type I	Estrogen receptor α	17b-estradiol
NR3A2/ESR2	Type I	Estrogen receptor β	17b-estradiol
NR3C1/GR	Type I	Glucocorticoid receptor	Cortisol
NR3C2/MR	Type I	Mineralocorticoid receptor	Aldosterone
NR3C3/PGR	Type I	Progesterone receptor	Progesterone
NR3C4/AR	Type I	Androgen receptor	Dihydrotestosterone
NR1A1/THRA	Type II	Thyroid hormone receptor α	Triiodothyronine
NR1A2/THRB	Type II	Thyroid hormone receptor β	Triiodothyronine
NR1B1/RARA	Type II	Retinoic acid receptor α	All trans retinoic acid
NR1B2/RARB	Type II	Retinoic acid receptor β	All trans retinoic acid
NR1B3/RARG	Type II	Retinoic acid receptor gamma	All trans retinoic acid
NR1C1/PPARA	Type II	Peroxisome proliferator-activated receptor α	Long-chain fatty acids
NR1C2/PPARD	Type II	Peroxisome proliferator-activated receptor delta	Fatty acid/eicosanoids
NR1C3/PPARG	Type II	Peroxisome proliferator-activated receptor gamma	15d-PGJ2
NR1H2/LXRB	Type II	Liver × Receptor β	Oxysterols
NR1H3/LXRA	Type II	Liver × Receptor α	Oxysterols
NR1H4/FXR	Type II	Bile acid receptor	Bile acids
NR1I1/VDR	Type II	Vitamin D receptor	1,25-dihydroxyvitamin D3
NR1I2/PXR	Type II	Pregnane × Receptor	Xenobiotics/bile acids
NR1I3/CAR	Type II	Constitutive androstane receptor	Constitutive; xenobiotics
NR2B1/RXRA	Type II	Retinoic acid receptor α	9-cis-retinoic acid
NR2B2/RXRB	Type II	Retinoic acid receptor β	9-cis-retinoic acid
NR2B3/RXRG	Type II	Retinoic acid receptor gamma	9-cis-retinoic acid
NR0B1/DAX1	Orphan	DSS-AHC critical region on X	No established ligand
NR0B2/SHP	Orphan	Small heterodimer partner	No established ligand
NR2C1/TR2	Orphan	NR subfamily 2 group C member 1	No established ligand
NR2C2/TR4	Orphan	NR subfamily 2 group C member 2	No established ligand
NR2E1/TLL (TLX)	Orphan	Protein tailless homolog	No established ligand
NR2E3/PNR	Orphan	Photoreceptor-specific NR	No established ligand
NR2F1/COUP-TFI	Orphan	COUP transcription factor 1	No established ligand
NR2F2/COUP-TFII	Orphan	COUP transcription factor 2	No established ligand
NR2F6/EAR2	Orphan	v-ErbA-related protein 2	No established ligand
NR4A1/NUR77	Orphan	NUR77	No established ligand
NR4A2/NURR1	Orphan	NURR1	No established ligand
NR4A3/MINOR	Orphan	Mitogen-induced nuclear orphan receptor	No established ligand
NR3B1/ESRRA	Orphan	Estrogen-related receptor α	No established ligand
NR3B2/ESRRB	Orphan	Estrogen-related receptor β	No established ligand
NR3B3/ESRRG	Orphan	Estrogen-related receptor gamma	No established ligand
NR6A1/RTR	Orphan	Retinoid receptor-related testis-specific receptor	No established ligand
NR1D1/Rev-ErbAalpha	Adop. orphan	REVERBα	Heme
NR1D2/Rev-ErbAbeta	Adop. orphan	REVERBβ	Heme
NR1F1/RORA	Adop. orphan	Retinoid-related orphan receptor α	Oxysterols
NR1F2/RORB	Adop. orphan	Retinoid-related orphan receptor β	Oxysterols
NR1F3/RORC	Adop. orphan	Retinoid-related orphan receptor gamma	Oxysterols
NR2A1/HNF4A	Adop. orphan	Hepatocyte nuclear factor 4 α	Fatty acids
NR2A2/HNF4G	Adop. orphan	Hepatocyte nuclear factor 4 gamma	Fatty acids
NR5A1/SF-1	Adop. orphan	Steroidogenic factor-1	Phospholipids
NR5A2/LRH-1	Adop. orphan	Liver receptor homolog-1	Phospholipids

Abbreviations: Adop., adopted; NR, nuclear receptor.

The NR superfamily consists of subgroups (NR0-NR6) based on phylogenetic relationships and structural properties. The DNA-binding domain (DBD) is highly conserved, being composed of 2 zinc fingers that allow binding to variously spaced and orientated hexameric motifs. Only 1 NR, NR0B2/SHP, lacks a DBD and instead functions as a coregulator for other DNA-bound NRs (3). By contrast, NR ligand-binding domains (LBDs) show significant diversity. The LBD of steroid hormone type I NRs bind ligands with high affinity, whereas LBDs of type II NRs show lower affinity for a wider diversity of ligands. LBDs from both type I and type II NRs respond to ligand by shifting the conformational status to obscure corepressor interaction domains and to expose coactivator interaction domains. The interactions between DBD and LBD have also been demonstrated and potentially allow for allosteric interactions to shape transcriptional effects (4-6).

Finally, orphan NRs either lack known endogenous ligands and/or have a structurally diminished LBD and may interact with other NRs either through overlapping binding at regulatory regions or through convergent yet nonoverlapping binding at the same target genes. Recent studies have identified profound cellular impacts for type II (independent of ligand) and orphan receptors including during zygote development (7, 8), embryogenesis (9), and tissue development (10-12), which reflect earlier studies from multiple aspects of tumor biology (13-17). The boundaries between type II and orphan NRs are not completely rigid, as in recent years ligands have been identified for orphan NRs, giving rise to the concept of adopted orphan NRs (18-20) (Table 1).

Coregulator complexes associate with NRs and exert diverse functions, including modifying histone states and other epigenomic events, as well as remodeling nucleosome positions. These coregulatory complexes also function to promote (when activating) or limit the spatial links between NRs at enhancers with the Mediator complex (21) and the general transcription machinery at the transcription start site. Therefore, coregulator dynamic interactions with NRs are reflected in the underlying changes to the epigenome and chromatin conformation. Given that type II NRs have a larger LBD than type I NRs and also have a more pronounced nuclear residence independent of ligand, the shift in cofactor interactions on type II NRs potentially exerts a more nuanced impact on gene expression (22-24).

Collectively, therefore, NRs function as an integrated signaling axis, or conduit, that combines extracellular and cytoplasmic signals to regulate the transcriptome and govern cell fate decisions [reviewed in (25)]. To exert these collective actions, there are multiple levels of NR crosstalk. In turn, many of these crosstalk mechanisms are variously targeted across hormone-dependent cancers.

## The Carcinogenic Impact of Altered NRs Interactions With Coregulators

Multiple NRs are expressed in the epithelial and other cell types of the mammary gland, prostate, ovaries, and endometrial tissues, and their functions are disrupted in the respective cancers through both de novo mechanisms and acquired alterations in response to therapy. The disruptions to NR levels range from changes in receptor expression (26) to the impact of germline (27) and somatic genetic variants (28) to the altered interactions with coregulatory protein complexes (29).

There are well-established and impactful examples of how NR-coregulator interactions are disrupted across cancer. In BrCa, amplification of the coactivator NCOA3/AIB1 (amplified in breast cancer 1) enhances estrogen receptor (ER) α activity and contributes to resistance toward selective estrogen receptor modulators such as tamoxifen (30). Similarly, phosphorylation of MED1 by MAPK pathways enhances ERα coactivator recruitment even in the presence of tamoxifen and leads to therapy resistance (31). In acute promyelocytic leukemia, the promyelocytic leukemia–retinoic acid receptor (RAR)α fusion protein binds the corepressor NCOR2 with abnormally high affinity, thereby repressing differentiation genes even in the presence of physiological levels of the RARα ligand all trans retinoic acid (32, 33). In PCa, TMPRRS2-ERG is a frequent gene fusion product that sustains NR3C4/androgen receptor (AR) coregulator interactions, thereby altering gene expression (34). Together, these examples underscore how aberrant coregulator engagement can convert NRs from context-dependent regulators of cell differentiation into constitutive drivers of malignancy. However, defining the specific and potentially carcinogenic interactions of coregulators with NRs is a significant research challenge as these receptors interact dynamically with a large armory of functionally diverse multimeric protein complexes to control gene regulation.

There are compelling arguments that changes to the epigenome arising from altered NR-coregulator interactions are carcinogenic. For instance, therapy resistance in PCa can emerge rapidly (35), is reversible (36), and is not explained fully by the burden of genomic alterations (37). Again, in PCa, the epigenomic changes are driven in part by qualitative and quantitative changes of AR interactions with pioneer factors (38), coregulators (29, 39-42), and other transcription factors (TFs) (40, 43-45). These altered AR interactions reshape the portfolio of accessible enhancers, resulting in changes to the transcriptional landscape (43, 46). As a result, AR signaling is altered or “rewired,” thereby disrupting normal AR control of prostate luminal lineage commitment [reviewed in (47)]. This rewiring promotes a heterogeneous tumor response to AR-targeted therapies (48). Indeed, PCa cells that are clinically resistant to AR-targeted therapies nonetheless display a transcriptional response toward these therapies (49), further supporting the idea that AR signaling is rewired, not lost.

### Coregulator Interchange Between NRs

The recruitment of coregulators is essential for NRs to function transcriptionally, and the mechanisms by which they are exchanged can shape their collective functions. The contrasting sizes of type I and type II NR LBDs allow for the possibility of different patterns of coregulator interactions that reshape crosstalk between NRs. For example, the normal mammary gland consists of multiple cell types, each with a different complement of NRs that allows for intra- and intercellular crosstalk. The lipid-sensing NR1C3/peroxisome proliferator-activated receptor gamma (PPARγ) (Table 1) in adipocytes (50) regulates the secretion of adipokines that in turn regulate hormone responses mediated by ERα in adjacent luminal epithelial cells (51). This crosstalk between ERα and PPARγ has been suggested as a chemotherapeutic strategy (52-59), although clinical translation has proved equivocal (57), suggesting that the prodifferentiation actions arising from the PPARγ and ERα crosstalk are corrupted in BrCa (57, 58).

A further explanation for the crosstalk between PPARγ and ERα is through commonalities and differences in coregulator recruitment by ERα and PPARγ. Specifically, the ERα LBD is ∼250 amino acids and has a tightly regulated response to 17β-estradiol in the low nM range (60). By contrast, the LBD of PPARγ is approximately twice as large as ERα (∼1300 Å^3^ vs ∼450-550 Å^3^), allowing a more diverse array of endogenous and synthetic ligands to bind (61). In short, ERα binds ligand with high affinity, leading to rapid transcriptional responses, whereas PPARγ binds a diverse range of lipid ligands in a more spacious pocket (61-65). Ligand activation of PPARγ allows a more nuanced recruitment of coregulators to fine-tune gene expression. As a consequence, PPARγ can cycle through intermediate (although weaker) cofactor accessible conformations (64), including not only with classical coactivators (66) and corepressors (67) but also with PPARγ exclusive coregulators such as PGC-1α (68) and tissue-specific partners such as PRDM16 (66, 69). A potential impact of these altered coregulator interactions between ERα and PPARγ is seen in the sequestration of GATA3 to genes targeted by these receptors (70-72).

A direct proteomic comparison of ERα and PPARγ interactions with coregulators has yet to be undertaken to confirm these differences in recruitment. Insights into how NR LBDs interact with coregulators are illustrated by their alterations in cancer. In BrCa *ESR1* mutations associate with endocrine therapy in a cluster of helix 12 at the coactivator interface (73). PPARγ is amplified and upregulated (74) in PCa and BrCa cohorts (75) but is not commonly mutated (and it is not commonly targeted with therapies), and therefore PPARγ expression changes and lack of mutations may also reflect more nuanced functions of PPARγ in cancer cells, for example with roles in differentiation and cell metabolism.

Given that BrCa, PCa, and other hormone-dependent cancers express multiple NRs and that this catalog includes type I, type II, and orphan receptors, then it is quite likely there are multiple other examples of how changes in expression and mutational status shape how coregulators are interchanged between NRs and thereby impact the local epigenome.

### The Choreography of NR Proteomic Interactions

NRs display a diverse array of protein interactions. Pioneer factors facilitate NR binding (76, 77), and subsequently multiple coregulator complexes assemble in a coordinated manner to govern transcriptional programs. The altered cycling of different complexes and their impact on transcription represent another mechanism by which NR signaling is altered.

#### Rapid coregulator and BRM-associated factor component interactions with NRs

The concept of assisted loading was developed to explain how rapid and dynamic binding of glucocorticoud receptor (NR3C1/GR) and cofactors can facilitate other NRs to bind. Rapid GR binding initiates chromatin remodeling and promotes ERα binding at the same genomic site (78). Expanding these studies to mammary epithelial studies also demonstrates GR activation modulates chromatin structure at specific enhancers that are then bound by ERα. Interestingly, the process also works in reverse, in that ERα can prime sites for GR binding, demonstrating reciprocal, assisted-loading behavior at subsets of enhancers. The same mechanism in the liver allows GR to dictate specific TF access, including PPARα (79).

The assisted loading framework has been extended to PCa in the context of AR and GR crosstalk, which is clinically significant in therapy-resistant PCa (80). Specifically, AR activation significantly expands GR chromatin occupancy, enabling GR to bind previously inaccessible enhancer regions (81). Importantly, SMARCA4, an ATP-dependent chromatin remodeling component in the SWI/SNF-related BRG1/BRM-associated factor (BAF) chromatin remodeling complex, was shown to promote AR-mediated assisted loading, reflecting earlier studies (82). Interestingly, coactivation of AR and GR produced unique transcriptional outputs that included the prominent upregulation of the orphan NR estrogen-related receptor gamma (NR3B3/ESRRG), which is associated with improved clinical outcome (81), again underscoring the crosstalk across the NR superfamily.

The role of the BAF complex to control NR functions and shape ERα transcriptional actions has also been investigated in BrCa. Specifically, ARID1A/BAF250 loss led to increased BRD4 occupancy and histone acetylation, reflecting a shift toward a more open, transcriptionally active chromatin state (83). This study demonstrated that corepressor–coactivator balance is not a static axis but is dynamically regulated by upstream chromatin remodelers such as the components of the BAF complexes, which gate access to transcriptional cofactors even in the presence of identical ligand conditions.

Early studies identified BAF components associated with the NCOR1 complex (84) and their requirement for chromatin remodeling as part of GR signaling (85). Recent proteomic approaches such as rapid immunoprecipitation mass spectrometry of endogenous protein (86) in BrCa and PCa have consistently identified BAF complex proteins significantly enriched in various NR complexes; ERα associated with other NRs (RARs/RXRs) and SMARCC1, SMARCA4, and SMARCA5 (87); AR with SMARCC1 and SMARCA4 (88); vitamin D receptor (VDR) with BAZ1A (which interacts with SMARCA5) (89); and NR1B3/RARγ with AR, SMARCC1, SMARCA4, and SMARCA5 (M.J.C., unpublished observations). Other proteomic approaches identified AR associated with SMARCC1 and SMARCA4 (62, 90). Collectively, these approaches suggest that ATP-dependent chromatin remodeling is a significant and common component of NR signaling complexes and that frequently more than 1 NR is present in these large complexes.

#### NR interactions with BAF complexes in mitosis and lineage commitment

BAF complexes control nucleosome positioning in various contexts, including transcription and DNA repair (91), and more recently have been shown to participate in the process of mitotic bookmarking. During mitosis, most proteins and epigenomic states are stripped away from chromatin, and therefore faithful transmission of transcriptional programs to daughter cells is a biological challenge. Cells meet this challenge by ensuring that during mitosis a few complexes remain chromatin bound to relay transgenerational epigenomic memory at promoter regions of genes required for lineage commitment programs (92). These protein complexes therefore bookmark lineage-critical gene regulatory regions (92-98). Bookmarking complexes include SMARCB1 (99) and SMARCA4 (100), although it is unclear which specific BAF complexes interact to ensure proper bookmarking (100). There is an emerging appreciation that type II and orphan NRs also participate in bookmarking; for example, estrogen-related receptor β (NR3B2/ESRRβ) has been identified in bookmarking complexes (101), as has the VDR (93).

How bookmarking by NRs and other TFs is underexplored, although it has been speculated that altered bookmarking events could be carcinogenic (98, 102, 103). In PCa, we demonstrated that RARγ exerts a mitotic bookmarking function to sustain luminal lineage programs. The RARγ complex associated with ESRRγ and several bookmarking factors, and in mitosis RARγ increased AR genomic binding. RARγ levels are commonly reduced in PCa (104), and restoring levels increased nucleosome-free regions at active enhancers enriched for H3K27ac and expanded the AR cistrome, allowing enzalutamide-regulated luminal transcriptional programs (M.J.C., unpublished observation).

The frequent enrichment of SMARC proteins in NR complexes and the nuclear residence of type II and orphan NRs independent of ligand both suggest that NR recruitment of bookmarking complexes may represent a further mechanism by which these receptors ensure correct lineage commitment in epithelial systems and that these processes may also be corrupted in hormone-dependent cancers.

#### Sustained coregulator interactions with NRs in the megatrans complex

The megatrans complex was identified by association with ERα and is able to guide it to unique enhancers. This process of guiding the ERα depended on the ligand-activated actions of RARα and RARγ in the complex (although their DNA binding was not required). In this manner, ERα and RARα/γ combined in the same large complex to augment not only ERα transcriptional actions (105) but also TFs such as GATA3 to generate a highly cooperative and sustained enhancer hub.

Interestingly, the same complex could be switched into a repressive one by ligand-activated GR, which evicted the ligand-bound RARα/γ and instead repressed ERα transcriptional activity by recruiting the NCOR2–HDAC3 corepressor complex (106). Such GR ligand-activated transrepression (107) helps to explain how the GR can reprogram transcription without changing the occupancy of other factors. This interplay of the megatrans with different type I and type II NRs, and possibly other TFs, suggests the possibility that this large complex could function as a sustained transcriptional complex. This interesting and seemingly important complex remains somewhat underexplored but has continued to receive significant attention from others examining how, for example, GR can crosstalk with other NRs in hormone-dependent cancers, as well as how it may relate to activation of a subset of superenhancers (108-111).

#### Pioneer factors, assisted loading, mitotic bookmarking, and megatrans: a functional continuum?

Somewhat like blind men with an elephant, it is unclear if these processes are a continuum of NR interactions with coregulator complexes or 4 distinct processes that independently impact NR biology. In part, this uncertainty arises as the experimental approaches used to define these mechanisms were different in terms of cells and treatments, as well as the technologies utilized. As a result, it is unclear if the proteins involved in assisted loading (which is rapid) could be stabilized through co-opted signaling events to form a more stable megatrans complex. Likewise, it is unclear whether, if assisted loading were to happen in mitosis, it can then initiate a bookmarking event. Similarities shared between the assisted loading, bookmarking, and megatrans complexes include 1 NR leading to the binding of another NR and show that this mechanism commonly requires BAF components as well as GATAs; the dynamic interplay reshapes enhancer landscapes, particularly during environmental or hormonal cues; and the functional consequence is to shape cell fate and lineage commitment decisions. There is also significant crossover with pioneer factor complexes, which often include FOXA1, GATAs, and BAF complex components.

**Figure 1.**
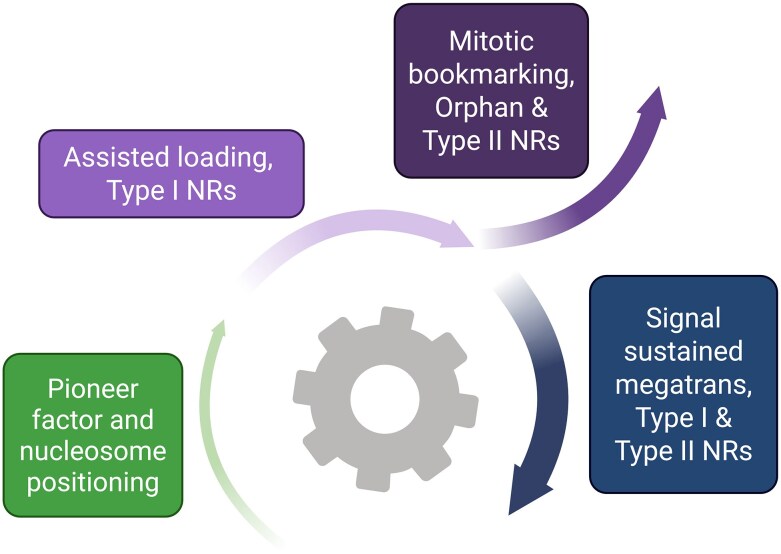
A proposed biological “ratchet” model of NR transcriptional functions. The model depicts a stepwise increase in transcriptional competence in which each state raises the likelihood of subsequent ones (“ratchet” behavior). Early steps are relatively deterministic, whereas later assembly becomes increasingly stochastic and signal dependent. Initially, pioneer factors and nucleosome positioning establish chromatin competence. Second, assisted loading enables rapid, cooperative recruitment whereby 1 NR facilitates another at shared regulatory elements. Third, mitotic bookmarking preserves open chromatin and NR occupancy across cell division, supporting inheritance of lineage programs. Finally, with sustained signaling, multivalent megatrans hubs form containing multiple NRs and stabilizing transcription. Boxes indicate NR classes with the strongest evidence at each step (type I: ligand-activated, eg, glucocorticoud receptor/androgen receptor (GR/AR); type II/orphan: retinoid X receptor heterodimers and estrogen receptor-related gamma). Arrows emphasize progression rather than strict linearity; states can coexist within a cell population. Created in BioRender. Abbreviation: NR, nuclear receptor.

In essence, these mechanisms may define a continuum (Fig. 1): pioneer complexes initiate chromatin remodeling and establish competence for NR binding; assisted loading can rapidly exploit this primed chromatin to enable perhaps more transient cooperation between NRs. If external signals are sustained, for example through the presence or autocrine generation of further NR ligands, this gives biological impetus to promote the formation of the multimeric megatrans complex that in turn stabilizes its interaction with chromatin. Arguably, in this context, bookmarking can be seen as a mechanism of preserving this process across mitosis to ensure inherited programming in daughter cells and perhaps also lead to assembly of megatrans complexes in daughter cells and sustained commitment to lineage decisions. Viewed through the lens of transcriptional bursting (112, 113), these mechanisms may represent a biological ratchet (Fig. 1) system that shifts target gene loci toward increased transcriptional competence, and in the context of cancer, this integrated mechanism allows for multiple stages of disruption in hormone-dependent cancers. Reflecting the concept that pioneer factors are more deterministic, mutations in PCa of the pioneer factor FOXA1 is more common than NRs (114) and have been shown to drive different oncogenic effects on downstream AR functions (115).

## NR Cistrome Interactions and Signaling Crosstalk

NR functions converge genomically at 2 levels. First, NR genomic binding frequently intersects at larger complex regulatory regions, which allows for integration of inputs into the control of gene expression (116). Second, target genes are frequently targeted by multiple NRs without genomic intersection. However, it is unclear what global mechanisms allow NRs to compete, cooperate, or act sequentially at the same or adjacent sites and how this may be corrupted in hormone-dependent cancers.

### Revisiting the 3-4-5 Rule

Key to addressing how NRs can converge is defining their genomic binding in terms of the orientation and spacing of hexameric *cis*-regulatory response elements. Within this context, workers in the 1990s developed the 3-4-5 rule (117-120) in which NR binding specificity was driven by the spacing between direct (DR), inverted (IR), or everted (ER) repeats of the hexameric motifs. The rationale was that the highly conserved DBDs could not be solely deterministic as they were so similar. Instead, hexamer spacing and orientation allowed NR complex specificity; for example, the AR binds to an IR spaced by 3 bp (IR3) and VDR binds to DR3.

This concept was intellectually elegant and ambitious given it was developed ahead of the release of the draft human genome in 2001 (121), but it began to fray with the application of cistromic approaches, which either were not able to map canonical motifs to predicted NRs or only identified them infrequently. Instead, NRs appear more frequently to be enriched at degenerate motifs, half-sites, or even regions without NR motifs.

Recent work (122) applying high-throughput HT-systematic evolution of ligands by exponential enrichment (SELEX) against highly degenerate DNA libraries of 20mer binding site (∼10 (12) unique sequences) has captured preferred DNA motifs for 45 human NRs. This approach dramatically increased the experimental space beyond the 3-4-5 rule to all 6 orientations (DR0-DR15, IR0-IR15, ER0-ER15) to capture DNA–protein interactomes and map receptor binding preferences exhaustively. Furthermore, the investigators met the significant bioinformatic challenges by designing a new algorithm (MinSeqFind) to extract the most informative sequence patterns beyond canonical motifs.

The MinSeqFind catalog revealed that type I NRs do indeed bind very strongly and exclusively to IR3 arranged motifs, but elsewhere binding across the NR interaction landscape was not driven solely by canonical half-site arrangements (DR, ER, IR) but instead a broader range of DNA configurations, including variable spacers, nonconventional motifs, and half-sites. Arguably, this is an example of how the early studies of steroid hormone type I NRs skewed the understanding of the whole family. Furthermore, even among type I NRs, fine-grained preferences emerged based on the spacer sequence and flanking regions, which shape DNA microstructure and electrostatic potential (122).

Numerous examples of NR heterodimerization and orientation suggest unsuspected convergent binding. For example, NR2F2/COUP-TFII could homodimerize and heterodimerize with RXR on DR1 motifs and showed affinity for noncanonical spacers such as DR6 and DR8, suggesting multiple routes to regulation such as either competitive binding or sequestration. However, the specific sites of NR binding preference were significantly impacted by cell type, suggesting that motif preference and chromatin states, coupled with coregulator expression, ultimately shape the NR binding landscape in a lineage-specific fashion. Thus, it appears that there is both a sophisticated alphabet of hexameric arrangements and a dramatic impact of the epigenome that combine to determine which NRs bind and where across the genome.

### Network Approaches to NRs Crosstalk

The SELEX approach demonstrated the importance of cell type to shape NR genomic interactions. Other investigators had also aimed to capture tissue-specific effects. In this manner, the Nuclear Receptor Signaling Atlas aimed to generate an atlas of NR functions in specific tissue types (123), and this effort has rolled into the larger Signaling Pathways Project (124). Similarly, the ENCODE project (125) included type I and type II NRs, which were investigated across different cell types through applying the same cell biology and genomic approaches coupled with harmonized and stringent bioinformatic analyses. The ENCODE cell models were classified in tiers. In this way, tier 1 cells were a small set of deeply profiled cell lines (GM12878, K562, and H1-hESC), which were studied across nearly all assays to maximize cross-comparability. Lower tiers encompassed a broader range of lineages and disease-relevant models that were analyzed in fewer assays, providing biological diversity while maintaining a core reference framework. NR chromatin immunoprecipitation sequencing in the tier 1 cell line K562 includes NR1H2/LXRβ NR2C1/TR2, NR2C2/TAK1, NR2F1/COUP-TF1, COUP-TFII, NR2F6/COUP-TFIII, PPARδ, GR, ESRRα, and ESRRβ, as well as factors known to interact with NRs such as SMARCA4. Likewise, there are multiple types of basal and signal-activated transcriptomic data in tier 1 cells, and therefore there is considerable potential to test NR cistrome intersections integrated with transcriptomic data (126). Likewise, integration of data in the Gene Expression Omnibus can also be revealing, although a significant impediment is the differences in experimental design (cell lines, receptor levels, time points, treatments, genetic manipulation, etc.) and inconsistent bioinformatic analyses; the CistromeDB consortia have addressed this latter point (127).

Perhaps the clearest demonstration of functional cistromic NR crosstalk was established in 2013 through a systems biology approach in BrCa (128). These investigators focused on MCF-7 cells and generated a comprehensive regulatory map of 24 NRs, 14 other TFs, and 6 chromatin markers alongside transcriptomic data. Furthermore, the authors employed BAC clones of green fluorescent protein (GFP)-tagged NRs such that expression was under the control of native promoters, ensuring relatively physiologically relevant expression and precise immunoprecipitation via GFP tag (a caveat to data interpretation was a concern over to what extent the GFP label disrupts intrinsic NR behavior). In this manner, over 200 000 significant binding sites were identified with ∼70% intersection between at least 2 factors, including so-called HOT regions, where more than 8 factors were significantly enriched in open chromatin near cancer-relevant genes (eg, *MYC*, *CCND1*).

Network approaches defined highly connected NR–TF circuits, including key hubs centered on ERα, RARs, GR, GATA3, and FOXA1 as well as potentially underexplored nodes focused on PPARδ, VDR, and THRα. Furthermore, ERα-regulated genes included other NRs (*RARA*, *RARG*, and *COUP-TFII*) whereas PPARγ, RARα, and VDR modulated ERα-dependent gene expression, reinforcing crosstalk and reciprocal regulatory loops. Finally, these relationships were clinically significant as PPARδ significantly intersected with RARα and RARγ and correlated with poorer prognosis in tumor samples, while RARs alone activity predicted favorable outcomes. Roles for RARα to promote/sustain luminal phenotypes in BrCa have recently also been established (129).

## Current Challenges and Future Opportunities

### Data Sparsity Impedes Biological Insights

Currently, there is a very large volume of cistromic, transcriptomic, proteomic, and other high-dimensional data focused on NR functions in hormone-dependent cancer cells. Indeed, some of the earliest examples of genomic technology implementation in biomedical research are centered on defining NR functions. Despite this progress and a large volume of data, understanding NR cancer biology remains limited, and the field still struggles to explain and predict

- How NR binding sites are chosen and distributed between distal vs proximal orientation to the target gene, what the qualitative motif differences across binding sites are, and how this shapes the magnitude and amplitude of gene transcription;
- Why the frequency and genomic distribution of NR binding sites differ so markedly between tumors and matched nonmalignant tissues;
- Why, within a single tumor type and stage, NR binding and NR-dependent gene expression vary so widely; and
- Why NR binding and gene regulations are strongly shaped by genomic ancestry (89, 130).

The question of enhancer distance to the target gene remains a challenging issue. For example, Hi-C studies of enhancer interactions reveal that the average contact distance of promoter to enhancer is ∼160 kb, each promoter associates with multiple enhancers, and enhancers can interact with multiple promoters, all often contained within the same topologically-associated domain; in this manner, this is a many-to-many problem. Furthermore, there is promoter-promoter looping, as well as intronic links to adjacent genes (131, 132).

The diversity of NR motifs revealed by high-throughput SELEX and similar approaches demonstrates that the composite motifs for multiple TFs are probably common and support an argument for competitive binding by multiple TFs to determine the final signal that emerges (133). That is, these data suggest widespread composite DNA binding motifs where the functional binding, and therefore the extent of crosstalk within and across TF families, is determined by the sum of overlapping binding TFs (133). These approaches have also encouraged the development of new bioinformatic methods to test the principles of how motifs significantly predict TF binding (134). More widely, composite motifs and convergent binding support a facilitator-type role for how NRs may function cooperatively.

An ongoing challenge is to explain how NR mutation and expression status explain observed behavior in tumors. Clearly, therapeutic resistance associates with acquisition of *ESR1* and *AR* mutations in BrCa and PCa, whereby the function of therapeutic antagonists are distorted. Outside of cancer, de novo mutations have been identified in type II NRs such as *LXRa* that disrupt metabolism (135). However, considering NRs as a family actually reveals they are significantly less mutated than predicted by chance in hormone-dependent cancers (136). NR expression levels vary within the same tumor type but for the type I NRs do not appear to explain clearly altered NR cistromes and gene expression (130). There is some evidence that specific type II NRs such as RARγ are significantly and commonly downregulated, perhaps because they exert growth restraint and promote differentiation (104).

A common focus then is to consider the mutational status and expression levels of pioneer factors and coregulators, which have emerged as highly prominent in cancer and functionally impact NR cistromes and transcriptomes (115). Again, there are multiple pioneer factors and coregulators, and their biological functions are often nuanced. Coactivators like NCOA3/SRC-3 can act as repressors in certain contexts [reviewed in (137)], and corepressors like NCOR2 can promote/stabilize transcription (29).

Data sparsity impedes developing a more complete understanding of how pioneer factors and coregulators shape NR functions. This is illustrated by the type I NRs like AR and ERα that have justifiably been profiled extensively in human and murine systems, given their therapeutic centrality, resulting in hundreds of AR and ERα cistromic datasets publicly deposited in the Gene Expression Omnibus. That said, other NRs are often more highly altered by mutation and expression in the same hormone-dependent cancers. For example, in TCGA-BRCA, *ESR1* is altered by mutation or expression in ∼10% of tumors, whereas other NRs (eg, *NR5A2/LRH-1*, *ESRRG*, *NR1I3/CAR*) are more frequently altered, and only a handful of cistromic datasets exist for these receptors across all cancers. Similar imbalances occur in PCa and other tumors. In practice, the NR field is data-rich for a few receptors, coregulators, and cell contexts and data-sparse for the rest. This knowledge gap is set to increase with the application of single-cell and spatial technologies.

### Experimental and Bioinformatic Challenges

Current experimental approaches to understanding NRs are limiting. Like Schrödinger's cat, the NR-chromatin interactions are indeterminate until observed, and each assay collapses a different slice of the uncertainty. Chromatin immunoprecipitation sequencing and CUT&RUN map NR chromatin occupancy but not the composition of the complex. Although CUT&RUN for histone modifications at single-cell resolution has been developed (138), similar approaches for TF binding remain elusive and therefore genomic associations are the average of all cells in the population. Finally, GIGGLE (139) and similar bioinformatic methods attempt to demonstrate the overlap of NR with other cistromes but again do not offer locus-specific resolution. Conversely, rapid immunoprecipitation mass spectrometry of endogenous protein and other proteomic approaches identify the proteins associated with the receptor yet lose genomic coordinates. Recent loci-specific proteomic approaches offer the possibility of addressing this (140), although whether they will function for NRs is uncertain.

Finally, a range of experimental design and bioinformatic challenges compound data sparseness. There is little consensus on the importance of biological triplicates for many genomic experiments; this is surprising as MIAME-compliant protocols were standardized for microarray experiments in 2001 (141). Likewise, there is a need for a strategic discussion of the application of short- vs long-read sequencing platforms. There are also bioinformatic considerations that are little discussed, namely, the use of the human pangenome reference for alignment for cells of different ancestry (142); how the union or intersection of replicates is considered in cistrome studies and how that impacts false discovery; the merits of motif enrichment tools; and how peaks are intersected, visualized, and annotated to genes. Without discussion and harmonization of these aspects, it will be hard to reduce apparent variability not explained by biology.

### The Opportunity for the NuRome

Previous researchers (143) as well as the Nuclear Receptor Signaling Atlas project aimed to apply unbiased approaches to capture NR signaling, which can be termed the “NuRome.” Despite falling sequencing costs, it remains infeasible to study all NRs in large panels of hormone-dependent cancer cell lines, with every treatment and genetic perturbation. As a result, NR studies will need to be prioritized in terms of experimental approaches and treatments as well as cells and genomic ancestries. Advances in genomic and CRISPR technologies as well as in mechanistic understanding provide a timely opportunity to define the NuRome in hormone-dependent cancers.

The biological ratchet model (Fig. 1) provides a useful prioritization framework to define the NuRome. In this model, pioneer factors and BAF components establish chromatin competency for subsequent NR signaling events that cooperatively participate in either assisted loading or bookmarking processes that, if either sustained or convergent, stabilize transcriptional hubs. Initiating chromatin remodeling events are shaped by pioneer factor occupancy and nucleosome positioning and are likely more deterministic, whereas downstream complex assembly and NR-driven transcription are more stochastic, including signal-dependent events and cell context.

**Figure 2.**
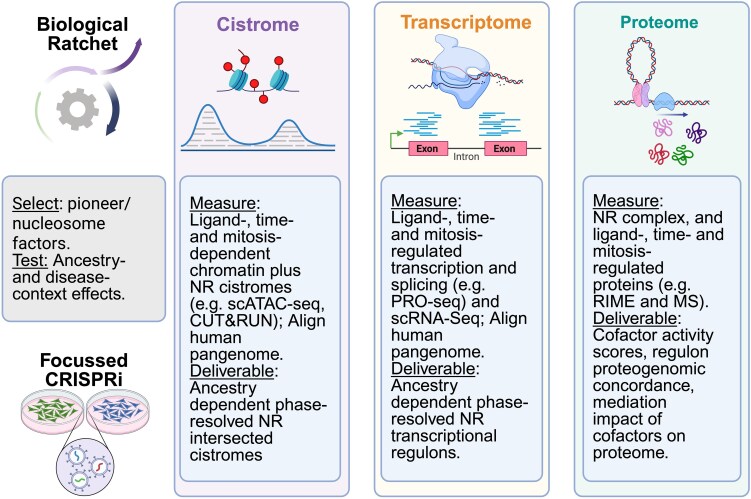
The NuRome workflow: a systems framework to measure NR crosstalk. The schematic summarizes a hierarchical pipeline that prioritizes determinants of NR action and iterates from discovery to prediction. Top left: biological ratchet. Pioneer factors and nucleosome-positioning complexes establish chromatin competence; NRs then engage in assisted loading, mitotic bookmarking, and, under sustained signaling, stabilize the multivalent megatrans hub. Middle: factor prioritization. Cross-cancer dependency resources (eg, depmap loss-of-function screens) nominate pioneer/BAF components with the strongest fitness effects in sensitive vs resistant models. Bottom left: focused perturbation. Targeted CRISPRi/KO perturbs nominated factors to test causal impact on enhancer states, NR occupancy, and functional impacts. Cistrome panel: measure effect of perturbations on ancestry-, ligand-, time-, and mitosis-dependent chromatin states (eg, ChromHMM) and select NR binding (eg, androgen receptor [AR] and commonly altered in prostate cancer) using (sc)ATAC-seq, CUT&RUN/CUT&Tag. Generate enhancer activity and motif features (eg, position weight matrices and overlapping-site density/index [ODI]). Transcriptome panel: quantify nascent and steady-state output under the same conditions with precision nuclear run-on (PRO) sequencing (PRO-seq) for initiation/pausing and enhancer RNAs alongside bulk and single-cell RNA sequencing (eg, isoform/pangenome aware alignments) to determine regulons. Proteome panel: map complexes and downstream effectors: NR interactomes (eg, RIME, TurboID); global and phospho-proteomics (eg, TMT). Integration/analysis: enhancer to gene linking and statistical frameworks that partition variance across modules (nested cross-validated R^2^ to measure variance impact of components such as motifs/ODI and chromatin) and thereby apportion the contribution to transcription and RNA abundance. In this manner, the impact of deterministic pioneer and remodeling factors on chromatin and NR overlapping features alongside cofactor expression is measured on regulons and protein networks. The resulting multiomic NuRome model supports predictive tests of the biological ratchet and prioritizes interventions that reprogram NR-driven cell-fate decisions. Created in BioRender. Abbreviations: BAF, BRM-associated factor; NR, nuclear receptor; RIME, rapid immunoprecipitation mass spectrometry of endogenous protein; TMT, tandem mass tag.

Bioinformatic and genomic workflows grounded in this hierarchy would prioritize NR signaling events (Fig. 2). In the first instance, dependency data (144) identifies which pioneer factors and BAF components are most impactful and focused CRISPRi/KO screens in therapy-sensitive vs -resistant cell variants can test their effects on the epigenomic landscape and the cistrome of principal NRs (eg, AR in PCa) as well as the transcriptome and proteome.

Ultimately, integrating cistrome, transcriptome, and proteome with motif models and statistical frameworks that partition variance will establish how control of enhancer states drives NR(s) occupancy and shapes downstream programs that in combination form the NuRome. Inevitably, the application of large language models will massively enhance the speed of data integration with publicly available data. The promise of such a NuRome approach will be to generate predicative models of NR functions that test how a biological ratchet model can predict the function of enhancers to shape NR complex assembly and crosstalk to bring about coordinated gene expression programs that ultimately control cell fate decisions.

## Data Availability

This is a review, and there is no data.

## Current Affiliation

Moray J. Campbell is currently affiliated with Karmanos Cancer Institute, Department of Oncology, Wayne State University School of Medicine, Detroit, MI 48201, USA.

## References

[1] Paul J . Sir george beatson and the royal beatson memorial hospital. Med Hist. 1981;25(2):200‐201. 7012484 PMC1139017

[2] Huggins C, Stevens R, Hodges CV. Studies on prostatic cancer: II. The effects of castration on advanced carcinoma of the prostate gland. Arch Surg. 1941;43(2):209‐223.

[3] Lee HK, Lee YK, Park SH, et al Structure and expression of the orphan nuclear receptor SHP gene. J Biol Chem. 1998;273(23):14398‐14402. 9603951 10.1074/jbc.273.23.14398

[4] Chandra V, Wu D, Li S, Potluri N, Kim Y, Rastinejad F. The quaternary architecture of RARbeta-RXRalpha heterodimer facilitates domain-domain signal transmission. Nat Commun. 2017;8(1):868. 29021580 10.1038/s41467-017-00981-y PMC5636793

[5] Yu X, Yi P, Hamilton RA, et al Structural insights of transcriptionally active, full-length androgen receptor coactivator complexes. Mol Cell. 2020;79(5):812‐823 e4. 32668201 10.1016/j.molcel.2020.06.031 PMC7483370

[6] Helsen C, Dubois V, Verfaillie A, et al Evidence for DNA-binding domain--ligand-binding domain communications in the androgen receptor. Mol Cell Biol. 2012;32(15):3033‐3043. 22645304 10.1128/MCB.00151-12 PMC3434514

[7] Gassler J, Kobayashi W, Gaspar I, et al Zygotic genome activation by the totipotency pioneer factor Nr5a2. Science. 2022;378(6626):1305‐1315. 36423263 10.1126/science.abn7478

[8] Kobayashi W, Sappler AH, Bollschweiler D, et al Nucleosome-bound NR5A2 structure reveals pioneer factor mechanism by DNA minor groove anchor competition. Nat Struct Mol Biol. 2024;31(5):757‐766. 38409506 10.1038/s41594-024-01239-0 PMC11102866

[9] Paredes A, Justo-Mendez R, Jimenez-Blasco D, et al Gamma-linolenic acid in maternal milk drives cardiac metabolic maturation. Nature. 2023;618(7964):365‐373. 37225978 10.1038/s41586-023-06068-7

[10] Tierney MT, Polak L, Yang Y, et al Vitamin A resolves lineage plasticity to orchestrate stem cell lineage choices. Science. 2024;383(6687):eadi7342. 38452090 10.1126/science.adi7342 PMC11177320

[11] Aboseif SR, Dahiya R, Narayan P, Cunha GR. Effect of retinoic acid on prostatic development. Prostate. 1997;31(3):161‐167. 9167767 10.1002/(sici)1097-0045(19970515)31:3<161::aid-pros3>3.0.co;2-o

[12] Lohnes D, Kastner P, Dierich A, Mark M, LeMeur M, Chambon P. Function of retinoic acid receptor gamma in the mouse. Cell. 1993;73(4):643‐658. 8388780 10.1016/0092-8674(93)90246-m

[13] Wang J, Zou JX, Xue X, et al ROR-gamma drives androgen receptor expression and represents a therapeutic target in castration-resistant prostate cancer. Nat Med. 2016;22(5):488‐496. 27019329 10.1038/nm.4070 PMC5030109

[14] Haller F, Bieg M, Will R, et al Enhancer hijacking activates oncogenic transcription factor NR4A3 in acinic cell carcinomas of the salivary glands. Nat Commun. 2019;10(1):368. 30664630 10.1038/s41467-018-08069-x PMC6341107

[15] Kang MH, Choi H, Oshima M, et al Estrogen-related receptor gamma functions as a tumor suppressor in gastric cancer. Nat Commun. 2018;9(1):1920. 29765046 10.1038/s41467-018-04244-2 PMC5954140

[16] Yu G, Corn PG, Shen P, et al Retinoic acid receptor activation reduces metastatic prostate cancer bone lesions by blocking the endothelial-to-osteoblast transition. Cancer Res. 2022;82(17):3158‐3171. 35802768 10.1158/0008-5472.CAN-22-0170 PMC9444986

[17] Zhang FX, Xu P, Zhang LJ, et al RARgamma promotes the invasion and metastasis of thyroid carcinoma by activating the JAK1-STAT3-CD24/MMPs axis. Int Immunopharmacol. 2023;125(Pt A):111129. 37897947 10.1016/j.intimp.2023.111129

[18] Wang Y, Kumar N, Crumbley C, Griffin PR, Burris TP. A second class of nuclear receptors for oxysterols: regulation of RORalpha and RORgamma activity by 24S-hydroxycholesterol (cerebrosterol). Biochim Biophys Acta. 2010;1801(8):917‐923. 20211758 10.1016/j.bbalip.2010.02.012 PMC2886165

[19] Wisely GB, Miller AB, Davis RG, et al Hepatocyte nuclear factor 4 is a transcription factor that constitutively binds fatty acids. Structure. 2002;10(9):1225‐1234. 12220494 10.1016/s0969-2126(02)00829-8

[20] Yin L, Wu N, Curtin JC, et al Rev-erbalpha, a heme sensor that coordinates metabolic and circadian pathways. Science. 2007;318(5857):1786‐1789. 18006707 10.1126/science.1150179

[21] Kang YK, Guermah M, Yuan CX, Roeder RG. The TRAP/mediator coactivator complex interacts directly with estrogen receptors alpha and beta through the TRAP220 subunit and directly enhances estrogen receptor function in vitro. Proc Natl Acad Sci U S A. 2002;99(5):2642‐2647. 11867769 10.1073/pnas.261715899 PMC122401

[22] Mendoza-Parra MA, Walia M, Sankar M, Gronemeyer H. Dissecting the retinoid-induced differentiation of F9 embryonal stem cells by integrative genomics. Mol Syst Biol. 2011;7:538. 21988834 10.1038/msb.2011.73 PMC3261707

[23] Nettles KW, Greene GL. Ligand control of coregulator recruitment to nuclear receptors. Annu Rev Physiol. 2005;67:309‐333. 15709961 10.1146/annurev.physiol.66.032802.154710

[24] Weston AD, Blumberg B, Underhill TM. Active repression by unliganded retinoid receptors in development: less is sometimes more. J Cell Biol. 2003;161(2):223‐228. 12719467 10.1083/jcb.200211117 PMC2172895

[25] De Bosscher K, Desmet SJ, Clarisse D, Estebanez-Perpina E, Brunsveld L. Nuclear receptor crosstalk—defining the mechanisms for therapeutic innovation. Nat Rev Endocrinol. 2020;16(7):363‐377. 32303708 10.1038/s41574-020-0349-5

[26] Koivisto P, Kononen J, Palmberg C, et al Androgen receptor gene amplification: a possible molecular mechanism for androgen deprivation therapy failure in prostate cancer. Cancer Res. 1997;57(2):314‐319. 9000575

[27] Tjader NP, Beer AJ, Ramroop J, et al Association of ESR1 germline variants with TP53 somatic variants in breast tumors in a genome-wide study. Cancer Res Commun. 2024;4(6):1597‐1608. 38836758 10.1158/2767-9764.CRC-24-0026 PMC11210444

[28] Lin CA, Chica-Parrado MR, Unni N, et al ESR1 y537S and D538G mutations drive resistance to CDK4/6 inhibitors in estrogen receptor-positive breast cancer. Clin Cancer Res. 2025;31(9):1667‐1675. 39992682 10.1158/1078-0432.CCR-24-2307 PMC12045714

[29] Long MD, Jacobi JJ, Singh PK, et al Reduced NCOR2 expression accelerates androgen deprivation therapy failure in prostate cancer. Cell Rep. 2021;37(11):110109. 34910907 10.1016/j.celrep.2021.110109 PMC8889623

[30] Anzick SL, Kononen J, Walker RL, et al AIB1, a steroid receptor coactivator amplified in breast and ovarian cancer. Science. 1997;277(5328):965‐968. 9252329 10.1126/science.277.5328.965

[31] Cui J, Germer K, Wu T, et al Cross-talk between HER2 and MED1 regulates tamoxifen resistance of human breast cancer cells. Cancer Res. 2012;72(21):5625‐5634. 22964581 10.1158/0008-5472.CAN-12-1305 PMC4141533

[32] Grignani F, De Matteis S, Nervi C, et al Fusion proteins of the retinoic acid receptor-alpha recruit histone deacetylase in promyelocytic leukaemia. Nature. 1998;391(6669):815‐818. 9486655 10.1038/35901

[33] Lin RJ, Nagy L, Inoue S, Shao W, Miller WH Jr, Evans RM. Role of the histone deacetylase complex in acute promyelocytic leukaemia. Nature. 1998;391(6669):811‐814. 9486654 10.1038/35895

[34] Shah N, Kesten N, Font-Tello A, et al ERG-Mediated Coregulator Complex formation maintains androgen receptor signaling in prostate cancer. Cancer Res. 2020;80(21):4612‐4619. 32934023 10.1158/0008-5472.CAN-20-2044

[35] Mu P, Zhang Z, Benelli M, et al SOX2 promotes lineage plasticity and antiandrogen resistance in TP53- and RB1-deficient prostate cancer. Science. 2017;355(6320):84‐88. 28059768 10.1126/science.aah4307 PMC5247742

[36] Zhang A, Lau NA, Wong A, et al Concurrent targeting of HDAC and PI3 K to overcome phenotypic heterogeneity of castration-resistant and neuroendocrine prostate cancers. Cancer Res Commun. 2023;3(11):2358‐2374. 37823778 10.1158/2767-9764.CRC-23-0250 PMC10658857

[37] Jolly MK, Kulkarni P, Weninger K, Orban J, Levine H. Phenotypic plasticity, bet-hedging, and androgen independence in prostate cancer: role of non-genetic heterogeneity. Front Oncol. 2018;8:50. 29560343 10.3389/fonc.2018.00050 PMC5845637

[38] Han M, Li F, Zhang Y, et al FOXA2 drives lineage plasticity and KIT pathway activation in neuroendocrine prostate cancer. Cancer Cell. 2022;40(11):1306‐1323 e8. 36332622 10.1016/j.ccell.2022.10.011

[39] Abida W, Cyrta J, Heller G, et al Genomic correlates of clinical outcome in advanced prostate cancer. Proc Natl Acad Sci U S A. 2019;116(23):11428‐11436. 31061129 10.1073/pnas.1902651116 PMC6561293

[40] Armenia J, Wankowicz SAM, Liu D, et al The long tail of oncogenic drivers in prostate cancer. Nat Genet. 2018;50(5):645‐651. 29610475 10.1038/s41588-018-0078-z PMC6107367

[41] Poluben L, Nouri M, Liang J, et al Increased nuclear factor I-mediated chromatin access drives transition to androgen receptor splice variant dependence in prostate cancer. Cell Rep. 2025;44(1):115089. 39709604 10.1016/j.celrep.2024.115089 PMC11921039

[42] Sardar S, McNair CM, Ravindranath L, et al AR coactivators, CBP/p300, are critical mediators of DNA repair in prostate cancer. Oncogene. 2024;43(43):3197‐3213. 39266679 10.1038/s41388-024-03148-4 PMC11493679

[43] Chan JM, Zaidi S, Love JR, et al Lineage plasticity in prostate cancer depends on JAK/STAT inflammatory signaling. Science. 2022;377(6611):1180‐1191. 35981096 10.1126/science.abn0478 PMC9653178

[44] Rotinen M, You S, Yang J, et al ONECUT2 is a targetable master regulator of lethal prostate cancer that suppresses the androgen axis. Nat Med. 2018;24(12):1887‐1898. 30478421 10.1038/s41591-018-0241-1 PMC6614557

[45] Shah N, Wang P, Wongvipat J, et al Regulation of the glucocorticoid receptor via a BET-dependent enhancer drives antiandrogen resistance in prostate cancer. Elife. 2017;6:e27861. 28891793 10.7554/eLife.27861 PMC5593504

[46] Shrestha R, Chesner LN, Zhang M, et al An atlas of accessible chromatin in advanced prostate cancer reveals the epigenetic evolution during tumor progression. Cancer Res. 2024;84(18):3086‐3100. 38990734 10.1158/0008-5472.CAN-24-0890 PMC12248187

[47] Ellis L . Understanding cancer lineage plasticity: reversing therapeutic resistance in metastatic prostate cancer. Pharmacogenomics. 2017;18(7):597‐600. 28468521 10.2217/pgs-2017-0039

[48] Romero R, Chu T, Gonzalez Robles TJ, et al The neuroendocrine transition in prostate cancer is dynamic and dependent on ASCL1. Nat Cancer. 2024;5(11):1641‐1659. 39394434 10.1038/s43018-024-00838-6 PMC11584404

[49] Westbrook TC, Guan X, Rodansky E, et al Transcriptional profiling of matched patient biopsies clarifies molecular determinants of enzalutamide-induced lineage plasticity. Nat Commun. 2022;13(1):5345. 36109521 10.1038/s41467-022-32701-6 PMC9477876

[50] Zhou F, Ouyang Y, Miao Y. Peroxisome proliferator-activated receptor gamma regulates genes involved in milk fat synthesis in mammary epithelial cells of water Buffalo. Anim Sci J. 2021;92(1):e13537. 33682250 10.1111/asj.13537

[51] Ardenkjaer-Skinnerup J, Saar D, Petersen PSS, et al PPARgamma antagonists induce aromatase transcription in adipose tissue cultures. Biochem Pharmacol. 2024;222:116095. 38423186 10.1016/j.bcp.2024.116095

[52] Knower KC, Chand AL, Eriksson N, et al Distinct nuclear receptor expression in stroma adjacent to breast tumors. Breast Cancer Res Treat. 2013;142(1):211‐223. 24122391 10.1007/s10549-013-2716-6

[53] Mehta RG, Peng X, Roy S, et al PPARgamma antagonist GW9662 induces functional estrogen receptor in mouse mammary organ culture: potential translational significance. Mol Cell Biochem. 2013;372(1–2):249‐256. 23001870 10.1007/s11010-012-1466-9

[54] Yaacob NS, Nasir R, Norazmi MN. Influence of 17beta-estradiol on 15-deoxy-delta12,14 prostaglandin J2 -induced apoptosis in MCF-7 and MDA-MB-231 cells. Asian Pac J Cancer Prev. 2013;14(11):6761‐6767. 24377602 10.7314/apjcp.2013.14.11.6761

[55] Elstner E, Muller C, Koshizuka K, et al Ligands for peroxisome proliferator-activated receptorgamma and retinoic acid receptor inhibit growth and induce apoptosis of human breast cancer cells in vitro and in BNX mice. Proc Natl Acad Sci U S A. 1998;95(15):8806‐8811. 9671760 10.1073/pnas.95.15.8806 PMC21158

[56] Mueller E, Sarraf P, Tontonoz P, et al Terminal differentiation of human breast cancer through PPAR gamma. Mol Cell. 1998;1(3):465‐470. 9660931 10.1016/s1097-2765(00)80047-7

[57] Loo SY, Syn NL, Koh AP, et al Epigenetic derepression converts PPARgamma into a druggable target in triple-negative and endocrine-resistant breast cancers. Cell Death Discov. 2021;7(1):265. 34580286 10.1038/s41420-021-00635-5 PMC8476547

[58] Hamel KM, King CT, Cavalier MB, et al Breast cancer-stromal interactions: adipose-derived stromal/stem cell age and cancer subtype mediated remodeling. Stem Cells Dev. 2022;31(19–20):604‐620. 35579936 10.1089/scd.2021.0279 PMC9595652

[59] Yang PB, Hou PP, Liu FY, et al Blocking PPARgamma interaction facilitates Nur77 interdiction of fatty acid uptake and suppresses breast cancer progression. Proc Natl Acad Sci U S A. 2020;117(44):27412‐27422. 33087562 10.1073/pnas.2002997117 PMC7959534

[60] Shiau AK, Barstad D, Loria PM, et al The structural basis of estrogen receptor/coactivator recognition and the antagonism of this interaction by tamoxifen. Cell. 1998;95(7):927‐937. 9875847 10.1016/s0092-8674(00)81717-1

[61] Nolte RT, Wisely GB, Westin S, et al Ligand binding and co-activator assembly of the peroxisome proliferator-activated receptor-gamma. Nature. 1998;395(6698):137‐143. 9744270 10.1038/25931

[62] Lam VQ, Zheng J, Griffin PR. Unique interactome network signatures for peroxisome proliferator-activated receptor gamma (PPARgamma) modulation by functional selective ligands. Mol Cell Proteomics. 2017;16(12):2098‐2110. 28972081 10.1074/mcp.RA117.000308 PMC5724174

[63] Brust R, Shang J, Fuhrmann J, et al A structural mechanism for directing corepressor-selective inverse agonism of PPARgamma. Nat Commun. 2018;9(1):4687. 30409975 10.1038/s41467-018-07133-w PMC6224492

[64] Chrisman IM, Nemetchek MD, de Vera IMS, et al Defining a conformational ensemble that directs activation of PPARgamma. Nat Commun. 2018;9(1):1794. 29728618 10.1038/s41467-018-04176-x PMC5935666

[65] Sabatino L, Ziccardi P, Cerchia C, et al Chiral phenoxyacetic acid analogues inhibit colon cancer cell proliferation acting as PPARgamma partial agonists. Sci Rep. 2019;9(1):5434. 30931956 10.1038/s41598-019-41765-2 PMC6443668

[66] Cho MC, Lee WS, Hong JT, et al 5-(3,5-Di-tert-butyl-4-hydroxybenzylidene) thiazolidine-2,4-dione modulates peroxisome proliferators-activated receptor gamma in 3T3-L1 adipocytes: roles as a PPARgamma ligand. Mol Cell Endocrinol. 2005;242(1–2):96‐102. 16171942 10.1016/j.mce.2005.08.005

[67] Battaglia S, Maguire O, Thorne JL, et al Elevated NCOR1 disrupts PPARalpha/gamma signaling in prostate cancer and forms a targetable epigenetic lesion. Carcinogenesis. 2010;31(9):1650‐1660. 20466759 10.1093/carcin/bgq086 PMC2930800

[68] Siddappa M, Wani SA, Long MD, et al Identification of transcription factor co-regulators that drive prostate cancer progression. Sci Rep. 2020;10(1):20332. 33230156 10.1038/s41598-020-77055-5 PMC7683598

[69] Kajimura S, Seale P, Tomaru T, et al Regulation of the brown and white fat gene programs through a PRDM16/CtBP transcriptional complex. Genes Dev. 2008;22(10):1397‐1409. 18483224 10.1101/gad.1666108 PMC2377193

[70] Metivier R, Penot G, Carmouche RP, et al Transcriptional complexes engaged by apo-estrogen receptor-alpha isoforms have divergent outcomes. EMBO J. 2004;23(18):3653‐3666. 15343269 10.1038/sj.emboj.7600377 PMC517616

[71] Wang L, Sun J, Yin Y, et al Transcriptional coregualtor NUPR1 maintains tamoxifen resistance in breast cancer cells. Cell Death Dis. 2021;12(2):149. 33542201 10.1038/s41419-021-03442-z PMC7862277

[72] Bi M, Zhang Z, Jiang YZ, et al Enhancer reprogramming driven by high-order assemblies of transcription factors promotes phenotypic plasticity and breast cancer endocrine resistance. Nat Cell Biol. 2020;22(6):701‐715. 32424275 10.1038/s41556-020-0514-z PMC7737911

[73] Najim O, Huizing M, Papadimitriou K, et al The prevalence of estrogen receptor-1 mutation in advanced breast cancer: the estrogen receptor one study (EROS1). Cancer Treat Res Commun. 2019;19:100123. 30826563 10.1016/j.ctarc.2019.100123

[74] Boretto C, Muzio G, Autelli R. PPARgamma antagonism as a new tool for preventing or overcoming endocrine resistance in luminal A breast cancers. Biomed Pharmacother. 2024;180:117461. 39326102 10.1016/j.biopha.2024.117461

[75] Kardos J, Chai S, Mose LE, et al Claudin-low bladder tumors are immune infiltrated and actively immune suppressed. JCI Insight. 2016;1(3):e85902. 27699256 10.1172/jci.insight.85902 PMC5033914

[76] Ji D, Shao C, Yu J, et al FOXA1 forms biomolecular condensates that unpack condensed chromatin to function as a pioneer factor. Mol Cell. 2024;84(2):244‐260 e7. 38101414 10.1016/j.molcel.2023.11.020

[77] Jiang G, Wang X, Sheng D, et al Cooperativity of co-factor NR2F2 with pioneer factors GATA3, FOXA1 in promoting ERalpha function. Theranostics. 2019;9(22):6501‐6516. 31588232 10.7150/thno.34874 PMC6771234

[78] Voss TC, Schiltz RL, Sung MH, et al Dynamic exchange at regulatory elements during chromatin remodeling underlies assisted loading mechanism. Cell. 2011;146(4):544‐554. 21835447 10.1016/j.cell.2011.07.006 PMC3210475

[79] Goldstein I, Baek S, Presman DM, Paakinaho V, Swinstead EE, Hager GL. Transcription factor assisted loading and enhancer dynamics dictate the hepatic fasting response. Genome Res. 2017;27(3):427‐439. 28031249 10.1101/gr.212175.116 PMC5340970

[80] Serritella AV, Shevrin D, Heath EI, et al Phase I/II trial of enzalutamide and mifepristone, a glucocorticoid receptor antagonist, for metastatic castration-resistant prostate cancer. Clin Cancer Res. 2022;28(8):1549‐1559. 35110415 10.1158/1078-0432.CCR-21-4049 PMC9012680

[81] Hiltunen J, Helminen L, Aaltonen N, et al Androgen receptor-mediated assisted loading of the glucocorticoid receptor modulates transcriptional responses in prostate cancer cells. Genome Res. 2025;35(8):1717‐1732. 40456604 10.1101/gr.280224.124 PMC12315708

[82] Paakinaho V, Swinstead EE, Presman DM, Grontved L, Hager GL. Meta-analysis of chromatin programming by steroid receptors. Cell Rep. 2019;28(13):3523‐3534 e2. 31553919 10.1016/j.celrep.2019.08.039 PMC6914262

[83] Nagarajan S, Rao SV, Sutton J, et al ARID1A influences HDAC1/BRD4 activity, intrinsic proliferative capacity and breast cancer treatment response. Nat Genet. 2020;52(2):187‐197. 31913353 10.1038/s41588-019-0541-5 PMC7116647

[84] Underhill C, Qutob MS, Yee SP, Torchia J. A novel nuclear receptor corepressor complex, N-CoR, contains components of the mammalian SWI/SNF complex and the corepressor KAP-1. J Biol Chem. 2000;275(51):40463‐40470. 11013263 10.1074/jbc.M007864200

[85] Fryer CJ, Archer TK. Chromatin remodelling by the glucocorticoid receptor requires the BRG1 complex. Nature. 1998;393(6680):88‐91. 9590696 10.1038/30032

[86] Mohammed H, D'Santos C, et al Endogenous purification reveals GREB1 as a key estrogen receptor regulatory factor. Cell Rep. 2013;3(2):342‐349. 23403292 10.1016/j.celrep.2013.01.010 PMC7116645

[87] Papachristou EK, Kishore K, Holding AN, et al A quantitative mass spectrometry-based approach to monitor the dynamics of endogenous chromatin-associated protein complexes. Nat Commun. 2018;9(1):2311. 29899353 10.1038/s41467-018-04619-5 PMC5998130

[88] Ruff SE, Vasilyev N, Nudler E, Logan SK, Garabedian MJ. PIM1 phosphorylation of the androgen receptor and 14-3-3 zeta regulates gene transcription in prostate cancer. Commun Biol. 2021;4(1):1221. 34697370 10.1038/s42003-021-02723-9 PMC8546101

[89] Siddappa M, Hussain S, Wani SA, et al African American prostate cancer displays quantitatively distinct vitamin D receptor cistrome-transcriptome relationships regulated by BAZ1A. Cancer Res Commun. 2023;3(4):621‐639. 37082578 10.1158/2767-9764.CRC-22-0389 PMC10112383

[90] Launonen KM, Paakinaho V, Sigismondo G, et al Chromatin-directed proteomics-identified network of endogenous androgen receptor in prostate cancer cells. Oncogene. 2021;40(27):4567‐4579. 34127815 10.1038/s41388-021-01887-2 PMC8266679

[91] Harrod A, Lane KA, Downs JA. The role of the SWI/SNF chromatin remodelling complex in the response to DNA double strand breaks. DNA Repair (Amst). 2020;93:102919. 33087260 10.1016/j.dnarep.2020.102919

[92] Ito K, Zaret KS. Maintaining transcriptional specificity through mitosis. Annu Rev Genomics Hum Genet. 2022;23:53‐71. 35440147 10.1146/annurev-genom-121321-094603 PMC9976632

[93] Kashyap J, Tyagi RK. Mitotic genome bookmarking by nuclear receptor VDR advocates transmission of cellular transcriptional memory to progeny cells. Exp Cell Res. 2022;417(1):113193. 35523304 10.1016/j.yexcr.2022.113193

[94] Wani SA, Campbell MJ. Genomic insights into non-steroidal nuclear receptors in prostate and breast cancer. Adv Exp Med Biol. 2022;1390:227‐239. 36107322 10.1007/978-3-031-11836-4_13

[95] Zraly CB, Zakkar A, Perez JH, et al The Drosophila MLR COMPASS complex is essential for programming cis-regulatory information and maintaining epigenetic memory during development. Nucleic Acids Res. 2020;48(7):3476‐3495. 32052053 10.1093/nar/gkaa082 PMC7144903

[96] Festuccia N, Owens N, Navarro P. Esrrb, an estrogen-related receptor involved in early development, pluripotency, and reprogramming. FEBS Lett. 2018;592(6):852‐877. 28834535 10.1002/1873-3468.12826

[97] Rana M, Dash AK, Ponnusamy K, Tyagi RK. Nuclear localization signal region in nuclear receptor PXR governs the receptor association with mitotic chromatin. Chromosome Res. 2018;26(4):255‐276. 30009337 10.1007/s10577-018-9583-2

[98] Chervova A, Molliex A, Baymaz HI, et al Mitotic bookmarking redundancy by nuclear receptors in pluripotent cells. Nat Struct Mol Biol. 2024;31(3):513‐522. 38196033 10.1038/s41594-023-01195-1 PMC10948359

[99] Zhu Z, Chen X, Guo A, et al Mitotic bookmarking by SWI/SNF subunits. Nature. 2023;618(7963):180‐187. 37225980 10.1038/s41586-023-06085-6 PMC10303083

[100] Mashtalir N, D'Avino AR, Michel BC, et al Modular organization and assembly of SWI/SNF family chromatin remodeling complexes. Cell. 2018;175(5):1272‐1288 e20. 30343899 10.1016/j.cell.2018.09.032 PMC6791824

[101] Festuccia N, Owens N, Chervova A, Dubois A, Navarro P. The combined action of Esrrb and Nr5a2 is essential for murine naive pluripotency. Development. 2021;148(17):dev199604. 34397088 10.1242/dev.199604 PMC8451941

[102] Zaidi SK, Nickerson JA, Imbalzano AN, Lian JB, Stein JL, Stein GS. Mitotic gene bookmarking: an epigenetic program to maintain normal and cancer phenotypes. Mol Cancer Res. 2018;16(11):1617‐1624. 30002192 10.1158/1541-7786.MCR-18-0415 PMC6214712

[103] Carceles-Cordon M, Orme JJ, Domingo-Domenech J, Rodriguez-Bravo V. The yin and yang of chromosomal instability in prostate cancer. Nat Rev Urol. 2024;21(6):357‐372. 38307951 10.1038/s41585-023-00845-9 PMC11156566

[104] Long MD, Singh PK, Russell JR, et al The miR-96 and RARgamma signaling axis governs androgen signaling and prostate cancer progression. Oncogene. 2019;38(3):421‐444. 30120411 10.1038/s41388-018-0450-6 PMC6336686

[105] Liu Z, Merkurjev D, Yang F, et al Enhancer activation requires trans-recruitment of a mega transcription factor complex. Cell. 2014;159(2):358‐373. 25303530 10.1016/j.cell.2014.08.027 PMC4465761

[106] Yang F, Ma Q, Liu Z, et al Glucocorticoid receptor:megaTrans switching mediates the repression of an ERalpha-regulated transcriptional program. Mol Cell. 2017;66(3):321‐331 e6. 28475868 10.1016/j.molcel.2017.03.019 PMC5510478

[107] Hua G, Ganti KP, Chambon P. Glucocorticoid-induced tethered transrepression requires SUMOylation of GR and formation of a SUMO-SMRT/NCoR1-HDAC3 repressing complex. Proc Natl Acad Sci U S A. 2016;113(5):E635‐E643. 26712006 10.1073/pnas.1522826113 PMC4747779

[108] Chen D, Parker TM, Bhat-Nakshatri P, et al Nonlinear relationship between chromatin accessibility and estradiol-regulated gene expression. Oncogene. 2021;40(7):1332‐1346. 33420376 10.1038/s41388-020-01607-2

[109] Mayayo-Peralta I, Gregoricchio S, Schuurman K, et al PAXIP1 and STAG2 converge to maintain 3D genome architecture and facilitate promoter/enhancer contacts to enable stress hormone-dependent transcription. Nucleic Acids Res. 2023;51(18):9576‐9593. 37070193 10.1093/nar/gkad267 PMC10570044

[110] West DC, Kocherginsky M, Tonsing-Carter EY, et al Discovery of a glucocorticoid receptor (GR) activity signature using selective GR antagonism in ER-negative breast cancer. Clin Cancer Res. 2018;24(14):3433‐3446. 29636357 10.1158/1078-0432.CCR-17-2793 PMC6530562

[111] White SM, Snyder MP, Yi C. Master lineage transcription factors anchor trans mega transcriptional complexes at highly accessible enhancer sites to promote long-range chromatin clustering and transcription of distal target genes. Nucleic Acids Res. 2021;49(21):12196‐12210. 34850122 10.1093/nar/gkab1105 PMC8643643

[112] Bartman CR, Hamagami N, Keller CA, et al Transcriptional burst initiation and polymerase pause release are key control points of transcriptional regulation. Mol Cell. 2019;73(3):519‐532 e4. 30554946 10.1016/j.molcel.2018.11.004 PMC6368450

[113] Jubb AW, Boyle S, Hume DA, Bickmore WA. Glucocorticoid receptor binding induces rapid and prolonged large-scale chromatin decompaction at multiple target loci. Cell Rep. 2017;21(11):3022‐3031. 29241532 10.1016/j.celrep.2017.11.053 PMC5745231

[114] Chakraborty G, Nandakumar S, Hirani R, et al The impact of PIK3R1 mutations and insulin-PI3K-glycolytic pathway regulation in prostate cancer. Clin Cancer Res. 2022;28(16):3603‐3617. 35670774 10.1158/1078-0432.CCR-21-4272 PMC9438279

[115] Eyunni S, Mannan R, Zhang Y, et al Divergent FOXA1 mutations drive prostate tumorigenesis and therapy-resistant cellular plasticity. Science. 2025;389(6764):eadv2367. 40570057 10.1126/science.adv2367 PMC12326538

[116] Mianesaz H, Goczi L, Nagy G, et al Genomic regions occupied by both RARalpha and VDR are involved in the convergence and cooperation of retinoid and vitamin D signaling pathways. Nucleic Acids Res. 2025;53(6):gkaf230. 40167329 10.1093/nar/gkaf230 PMC11959543

[117] Vivanco Ruiz MM, Bugge TH, Hirschmann P, Stunnenberg HG. Functional characterization of a natural retinoic acid responsive element. EMBO J. 1991;10(12):3829‐3838. 1657595 10.1002/j.1460-2075.1991.tb04952.x PMC453120

[118] Forman BM, Casanova J, Raaka BM, Ghysdael J, Samuels HH. Half-site spacing and orientation determines whether thyroid hormone and retinoic acid receptors and related factors bind to DNA response elements as monomers, homodimers, or heterodimers. Mol Endocrinol. 1992;6(3):429‐442. 1316541 10.1210/mend.6.3.1316541

[119] Kurokawa R, Yu VC, Naar A, et al Differential orientations of the DNA-binding domain and carboxy-terminal dimerization interface regulate binding site selection by nuclear receptor heterodimers. Genes Dev. 1993;7(7B):1423‐1435. 8392479 10.1101/gad.7.7b.1423

[120] Perlmann T, Rangarajan PN, Umesono K, Evans RM. Determinants for selective RAR and TR recognition of direct repeat HREs. Genes Dev. 1993;7(7B):1411‐1422. 8392478 10.1101/gad.7.7b.1411

[121] Lander ES, Linton LM, Birren B, et al Initial sequencing and analysis of the human genome. Nature. 2001;409(6822):860‐921. 11237011 10.1038/35057062

[122] Bhimsaria D, Rodriguez-Martinez JA, Mendez-Johnson JL, et al Hidden modes of DNA binding by human nuclear receptors. Nat Commun. 2023;14(1):4179. 37443151 10.1038/s41467-023-39577-0 PMC10345098

[123] Margolis RN, Evans RM, O'Malley BW, Consortium NA. The nuclear receptor signaling atlas: development of a functional atlas of nuclear receptors. Mol Endocrinol. 2005;19(10):2433‐2436. 16051673 10.1210/me.2004-0461

[124] Ochsner SA, Abraham D, Martin K, et al The Signaling Pathways Project, an integrated ‘omics knowledgebase for mammalian cellular signaling pathways. Sci Data. 2019;6(1):252. 31672983 10.1038/s41597-019-0193-4 PMC6823428

[125] Luo Y, Hitz BC, Gabdank I, et al New developments on the encyclopedia of DNA elements (ENCODE) data portal. Nucleic Acids Res. 2020;48(D1):D882‐D889. 31713622 10.1093/nar/gkz1062 PMC7061942

[126] Long MD, van den Berg PR, Russell JL, Singh PK, Battaglia S, Campbell MJ. Integrative genomic analysis in K562 chronic myelogenous leukemia cells reveals that proximal NCOR1 binding positively regulates genes that govern erythroid differentiation and Imatinib sensitivity. Nucleic Acids Res. 2015;43(15):7330‐7348. 26117541 10.1093/nar/gkv642 PMC4551916

[127] Zheng R, Wan C, Mei S, et al Cistrome Data Browser: expanded datasets and new tools for gene regulatory analysis. Nucleic Acids Res. 2019;47(D1):D729‐D735. 30462313 10.1093/nar/gky1094 PMC6324081

[128] Kittler R, Zhou J, Hua S, et al A comprehensive nuclear receptor network for breast cancer cells. Cell Rep. 2013;3(2):538‐551. 23375374 10.1016/j.celrep.2013.01.004

[129] Liang J, Yao X, Aouad P, et al ERalpha dysfunction caused by ESR1 mutations and therapeutic pressure promotes lineage plasticity in ER(+) breast cancer. Nat Cancer. 2025;6(2):357‐371. 39805955 10.1038/s43018-024-00898-8

[130] Berchuck JE, Adib E, Abou Alaiwi S, et al The prostate cancer androgen receptor cistrome in African American men associates with upregulation of lipid metabolism and immune response. Cancer Res. 2022;82(16):2848‐2859. 35731919 10.1158/0008-5472.CAN-21-3552 PMC9379363

[131] Jung I, Schmitt A, Diao Y, et al A compendium of promoter-centered long-range chromatin interactions in the human genome. Nat Genet. 2019;51(10):1442‐1449. 31501517 10.1038/s41588-019-0494-8 PMC6778519

[132] Schmitt AD, Hu M, Jung I, et al A compendium of chromatin contact maps reveals spatially active regions in the human genome. Cell Rep. 2016;17(8):2042‐2059. 27851967 10.1016/j.celrep.2016.10.061 PMC5478386

[133] Khetan S, Carroll BS, Bulyk ML. Multiple overlapping binding sites determine transcription factor occupancy. Nature. 2025;646(8086):1001‐1011. 40903577 10.1038/s41586-025-09472-3 PMC12447533

[134] Zhou Q, Alberto de la Paz J, Stanowick AD, Lin X, Morcos F. Characterizing DNA recognition preferences of transcription factors using global couplings and high-throughput sequencing. Nucleic Acids Res. 2025;53(12):gkaf592. 40598892 10.1093/nar/gkaf592 PMC12214022

[135] Lockhart SM, Muso M, Zvetkova I, et al Damaging mutations in liver X receptor-alpha are hepatotoxic and implicate cholesterol sensing in liver health. Nat Metab. 2024;6(10):1922‐1938. 39322746 10.1038/s42255-024-01126-4 PMC11496107

[136] Long MD, Campbell MJ. Pan-cancer analyses of the nuclear receptor superfamily. Nucl Receptor Res. 2015;2:101182. 27200367 10.11131/2015/101182 PMC4869537

[137] Li L, Deng CX, Chen Q. SRC-3, a steroid receptor coactivator: implication in cancer. Int J Mol Sci. 2021;22(9):4760. 33946224 10.3390/ijms22094760 PMC8124743

[138] Womersley HJ, Muliaditan D, DasGupta R, Cheow LF. Single-nucleus CUT&RUN elucidates the function of intrinsic and genomics-driven epigenetic heterogeneity in head and neck cancer progression. Genome Res. 2025;35(1):162‐177. 39622638 10.1101/gr.279105.124 PMC11789629

[139] Layer RM, Pedersen BS, DiSera T, Marth GT, Gertz J, Quinlan AR. GIGGLE: a search engine for large-scale integrated genome analysis. Nat Methods. 2018;15(2):123‐126. 29309061 10.1038/nmeth.4556 PMC5872823

[140] Cenik BK, Aoi Y, Iwanaszko M, et al TurboCas: a method for locus-specific labeling of genomic regions and isolating their associated protein interactome. Mol Cell. 2024;84(24):4929‐4944 e8. 39706164 10.1016/j.molcel.2024.11.007 PMC12327812

[141] Brazma A, Hingamp P, Quackenbush J, et al Minimum information about a microarray experiment (MIAME)-toward standards for microarray data. Nat Genet. 2001;29(4):365‐371. 11726920 10.1038/ng1201-365

[142] Liao WW, Asri M, Ebler J, et al A draft human pangenome reference. Nature. 2023;617(7960):312‐324. 37165242 10.1038/s41586-023-05896-x PMC10172123

[143] Carlberg C, Dunlop TW. An integrated biological approach to nuclear receptor signaling in physiological control and disease. Crit Rev Eukaryot Gene Expr. 2006;16(1):1‐22. 16584379 10.1615/critreveukargeneexpr.v16.i1.10

[144] Arafeh R, Shibue T, Dempster JM, Hahn WC, Vazquez F. The present and future of the cancer dependency map. Nat Rev Cancer. 2025;25(1):59‐73. 39468210 10.1038/s41568-024-00763-x

